# Distinct Particle Films Impacts on Olive Leaf Optical Properties and Plant Physiology

**DOI:** 10.3390/foods10061291

**Published:** 2021-06-04

**Authors:** Annalisa Rotondi, Lucia Morrone, Osvaldo Facini, Barbara Faccini, Giacomo Ferretti, Massimo Coltorti

**Affiliations:** 1 Institute for the Bioeconomy, Italian National Research Council, via P. Gobetti 101, 40129 Bologna, Italy; annalisa.rotondi@ibe.cnr.it (A.R.); osvaldo.facini@ibe.cnr.it (O.F.); 2 Department of Physics and Earth Science, University of Ferrara, via Saragat 1, 44122 Ferrara, Italy; fccbbr@unife.it (B.F.); frrgcm@unife.it (G.F.); massimo.coltorti@unife.it (M.C.)

**Keywords:** *Olea europaea*, kaolin, zeolitite, foliar treatments, sustainable agriculture, crop defense

## Abstract

The olive fruit fly is worldwide considered a major harmful pest of the olive agroecosystem. In Italy, the fruit fly infestation is traditionally countered by spraying chemical insecticides (e.g., dimethoate), but due to the recent ban of dimethoate by the Reg EU2019/1090 and the increasing awareness of consumers of food sustainability, the interest in developing chemical-free alternatives to pesticides, such as the use of particle-films, is rising. A field experiment was conducted to assess the effect of different particle films (kaolin-base and zeolitite-base) on leaf gas exchanges and leaf optical properties. Results showed that with the dust accumulation on the leaves’ surface, photosynthesis, stomatal conductance, transpiration and water use efficiency were significantly lower in kaolin-treated olive trees compared to those treated with zeolitite and to the control, while olive trees treated with zeolitite showed physiological parameters similar to the untreated plants. Microstructural differences of different particle film on the leaf and olive surfaces emerged by ESEM observations also influenced leaf optical properties. Oils produced by zeolitite-treated plants show higher intensities of gustatory and olfactory secondary flavors compared to kaolin and test oils.

## 1. Introduction

The olive fruit fly (*Bactrocera oleae*) is worldwide considered a major harmful pest of the olive agroecosystem. Under certain environmental conditions (high humidity and precipitations and temperature below 28–30 °C), the fruit fly is responsible for large infestations that seriously compromise olive yield and oil quality [1].

The many olives strongly attacked by flies produce oxidized oils with a reduced quantity of phenolic substances, which therefore are unlikely to live up to the EFSA health claim [2]. In Italy, the fruit fly infestation is traditionally countered by spraying chemical insecticides such as dimethoate (in integrated regime) or by applying organic formulations (organic farming) [3]. However, taking into account the recent ban of dimethoate [4] and the increasing awareness of consumers of food sustainability, the interest in developing natural and chemical-free alternatives to pesticides, such as organic agrochemicals or the use of geologic material as particle film, is rising [5].

Agronomic practices are also one of the keys to allow the development of extra virgin olive oil (EVOO) market niches, guaranteeing high and constant quality standards [6].

The spraying of “rock dust” (e.g., kaolin) as foliar treatment in organic agriculture to reduce the negative impact of environmental stresses and to protect fruits from insect pests is a well-established approach [7]. Kaolinite (Al_2_Si_2_O_5_(OH)_4_) is an aluminium–silicate clay mineral composed of a layered silicon-oxygen tetrahedron and a layered aluminium–oxygen octahedron [8,9]; the commercial term “kaolin” refers to a rock whose percentage of kaolinite is higher than 50% [10]. In kaolin, kaolinite is often associated with other minerals such as quartz, feldspar and various phyllosilicates (such as muscovite and illite) [11]. Contrary to other clay minerals, such as smectites, kaolinite is characterized by a relatively low cation exchange capacity (CEC) (0.38 meq/g) [12]. The size of kaolinite particles can reach a colloidal level after milling and grinding during mineral processing [13].

Similarly, natural zeolites represent another geologic material that can be used as particle films for crop protection [14].

Zeolites are crystalline aluminosilicates composed of a 3D framework of linked [SiO_4_]^4−^ and [AlO_4_]^5−^. The framework delimits open cavities in the form of channels and cages in which H_2_O molecules and extra-framework cations can be reversibly exchanged. The most important properties of zeolite minerals are (i) high cation-exchange capacity, (ii) reversible dehydration and (iii) molecular sieve. Nowadays, more than sixty types of natural zeolites have been described by researchers (http://www.iza-online.org/natural/default.htm (accessed on 15 March 2021)), each differing in terms of framework structure, mineral chemistry and ion exchange capacity, but only a few occur in sufficient amounts and purity to be considered as exploitable natural resources [15]. Among them, clinoptilolite is the most frequent and abundant sedimentary zeolite in nature, followed, in the order, by mordenite > chabazite > phillipsite > erionite [16]. Natural zeolites are often constituents of volcanic tuffs [17]; thus, the term “natural zeolites” is inappropriate from a geological perspective and it should be substituted by rocks or tuffs rich in zeolite. Analogously to kaolin, if the zeolite content is greater than 50%, the rock can be classified as “zeolitite”, specifying the main zeolite constituent (e.g., chabazitic-zeolitite) [16].

Chabazite zeolite (CHA), although less abundant than clinoptilolite, is particularly attractive for agricultural and industrial applications because of its very high CEC (3.84 meq/g) and easiness in sorption and subsequent release of NH_4_^+^ ions [18,19]. The “honeycomb” framework of zeolite minerals, together with their carbon dioxide sorption and heat stress reduction capacity, makes them suitable as leaf coating products. Furthermore, their reversible dehydration makes them effective against fungal disease and insect pests [14]. Zeolite tuffs are most commonly used in agricultural practices as a soil amendment and for improving the nitrogen use efficiency (NUE) by crops because of their high affinity with NH_4_^+^ ions [20,21]. Recently, Italian CHA-zeolitite was used as a soil amendment in a long-term field experiment [22,23,24]. Laboratory incubations highlighted the positive effects of CHA-zeolitite on soil N and C gaseous emissions and microbial biomass [25,26]. The same rock was used for removing N and Na from animal liquid manure and low-quality irrigation waters, with promising results [27,28,29].

Studies on the use of powders for contrasting olive fly are fairly recent, and showed that kaolin application on the olive fruit fly significantly reduced the percentage of infested olives [30,31].

Rumbos et al. [32] studied the insecticide potential of zeolite formulations against stored grain insects but, to the best of our knowledge, zeolite tuffs have not yet been studied as a defense tool against the olive fly.

Regions characterized by arid climate and low rainfall regimes are the most suitable for this technology due to the reduced temperature of the leaves and the wash-off risk for the particle films. High rainfall regimes may lead to the necessity of multiple applications, increasing the costs (for materials and manpower) and hence significantly decreasing the attractiveness of the methodology [33].

Besides the effectiveness against the fruit fly, it is crucial to understand if the particle films interfere with the physiological activity of the plants, as the literature shows contrasting evidence on this subject. Some authors reported that kaolin film causes a reduction in leaf temperature, transpiration and water use efficiency (WUE) in soybean plants [34], as well as in apple leaves [35]. Contrarily, Jifon and Syvertsen [36] reported that the WUE of the kaolin-treated citrus leaf was higher than untreated leaves because photosynthesis was increased without an increase in leaf transpiration. In apple trees, the lower leaf temperature of kaolin-treated plants increased photosynthesis and stomatal conductance [37].

As mentioned above, the effect of zeolitite particle film on plant physiology is mostly unknown due to its recent application in agriculture. Besides the reduction of heat stress, zeolitites may also be used to reduce water stress. The adsorption selectivity of zeolites for water is greater than any other minerals [38], leading to an adsorption capacity that may reach up to 30% of the zeolite weight without any volume modification, depending on the zeolite type [39]. Thanks to these properties, together with the relatively low-cost and high abundance, zeolite attractiveness for agricultural utilizations has recently risen, overcoming that of kaolin.

According to Reddy et al. [40], the application of particle films over the stomata is known to increase resistance to water vapor losses. Moreover, particulate sprays modify the leaf optical properties, increasing foliage reflectivity and modifying plant physiological processes such as photosynthesis, morphogenesis and water balance [41].

The olive leaves are covered by trichomes, which may directly influence the diffusion boundary layer of the leaf surface, increase leaf reflectance for all wavelengths of solar radiation between 400 and 300 nm and restrict radiation absorbance, resulting in a reduction of the leaf load [42]. 

The experiments presented here were carried out in order to test the effectiveness of different particle films in a cold and humid environment, typical of northern Italy, where the olive fly attack is increasingly worrying. Here, small-scale, high quality olive oil production is carried out on the Emilia-Romagna Appennine hillsides.

In addition, this study aims to evaluate and compare the effects of two different particle films (kaolin and zeolitite) on leaf optical properties, leaf gas exchange and on the incidence of the olive fruit fly attack. ESEM observations allowed us to investigate the microstructural differences of the particle film on leaf and olive surfaces. Olive fruit analyses and sensory characterization of olive oils produced by the different treatments were also performed, in order to establish if the influence of foliar application on the ecophysiological parameters could affect harvest quality.

## 2. Materials and Methods

### 2.1. Treatments and Sampling

The study was carried out in 15-year-old commercial olive (*Olea europeae*) cv Correggiolo plants located in Bologna hills (Italy). One third of the olive orchard was submitted to kaolin treatment (K), 1/3 to zeolitite treatment (Z) and in the last 1/3 of orchard no applications were made (T). Two olive trees for each thesis were chosen, four branches for each tree were marked in different cardinal points, and for each branch three leaves were sampled. Twenty-four leaves for each thesis were considered for physiological, optical, ESEM and color leaf measurements. The tested treatments were:

(1) K: foliar application of kaolin at a dosage of 3.0 kg/100 L of H_2_O;
(2) Z: foliar application of CHA-zeolitite at a dosage of 0.6 kg/100 L of H_2_O;
(3) T: control (untreated).

The kaolin and the CHA-zeolitite were supplied by Balco s.p.a company. The mineralogical composition of both products is reported in Supplementary Table S1.

The tested application dosages were chosen according to the guidelines provided by the producer. Kaolin and CHA-zeolitite were applied by covering the total foliage using a mounted sprayer (flow max 50 L/min, capacity 200 l-Idromeccanica Bertolini-Reggio Emilia Italy) equipped with a handgun sprayer and testing different nozzle diameters. The average particle size of both kaolin and CHA-zeolitite was 6–10 µm.

The foliar applications started at the beginning of the summer, when olive fruits were developed enough to be attacked by *Bactrocera oleae*, and applications were repeated approximately every 20 days (13 June, 3 July, 21 July, 17 August, 5–12–19–29 September 2019), the applications were repeated after heavy precipitations (September) to guarantee sufficient coverage until the end of the growing season. Conventional orchard agronomic practises, pruning and winter treatment based on Bordeaux mixture, were applied for all thesis. Environmental temperatures and rainfalls were monitored using a weather station IRDAM WST 7000 C (IRDAM SA, Yverdon-les-Bains, Suisse).

50 g of leaves were randomly sampled from each olive plant to carry out elemental and isotopic analysis of C and N to check for possible differences in C-N composition between the studied plants. Once in the laboratory, the leaves were washed with deionized water, dried for three days at 60 °C and then ground to a fine powder. Additionally, to gain information on the soil environment, soil samples from the first 0.3 m depth were collected using a manual auger (Eijkelkamp). To address spatial variability, three logs per plant were mixed to form a global sample; each one was then sieved at 5 mm and air-dried before further analyses.

### 2.2. Environmental Electronic Microscope (ESEM) Observations

Leaf and fruit samples treated with different particle films were collected during the study according to the methodology reported by Lanza and Di Serio [43]. Samples were observed by ESEM (Zeiss, EVO LS 10, Oberkochen, Germany).

**Figure 1:**
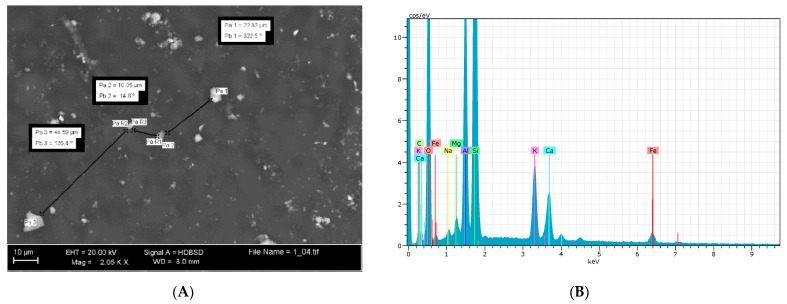
ESEM observations of CHA-zeolitite leaf coating to check the distribution protocol. (**A**) Measurements of the distance between CHA-zeolitite particles; (**B**) CHA-zeolitite particles’ composition by EDS-microanalysis.

To assure a homogeneous distribution of the particle films on the olive surface, preliminary observations were carried out by ESEM. Generally, obtaining good coverage is mandatory when using non-systemic products, such as zeolitite or kaolin. This is because only the “covered” areas of the canopy surface are protected [44]. To this aim, the droplet size distribution during atomization is very important because it affects the biological activity and the spray drift [45]. Study by Skuterud et al. [46] showed that, when applying contact products such as zeolitite, it is important to use fine (60 µm) or medium-sized (60–200 µm) droplets. The final coverage is also affected by the spray type: high application volumes can result in product run-off, which leads to considerable losses. On the other hand, low volume spraying leads to very poor coverage of the leaf surface and hence loss of efficacy [45]. Considering also the lower concentration of zeolite compared to the concentration of kaolin it was necessary to identify the right diameter of nozzles to guarantee a homogenous coating. This was achieved through several ESEM observations and measurements of the distance between the crystals (Figure 1A). These observations and measurements have confirmed that good coverage was achieved when spraying CHA-zeolitite utilizing a handgun sprayer with 0.2 mm diameter nozzles. These nozzles cause a dispersion of the product characterized by a distance among crystals under tenths of millimetres which is far smaller than the area interested by oviposition puncture of *Bactrocera oleae* (triangular slot of 1–1.5 millimetres long).

To establish the exact nature of the observed particles, semi-quantitative EDS (energy dispersive spectroscopy) microanalysis systems were carried out randomly to determine particles’ composition (Figure 1B).

### 2.3. Chemical Analysis on Leaves and Soil Samples

A total of 50 g of leaves and 500 g of soil samples were analysed for total C and N and the relative isotopic signature (δ^13^C and δ^15^N) with an Elementar Vario Micro Cube Elemental Analyzer (EA) in line with an ISOPRIME 100 Isotopic RatioMass Spectrometer (IRMS) operating in continuous-flow mode (Elementar Analysensysteme GmbH, Langenselbold, Germany). Soil samples were additionally processed for X-ray fluorescence (XRF) analysis on powder pellets, using a wavelength-dispersive automated ARL Advant’X spectrometer (Thermo Electron SA, Ecublens, Switzerland). The organic matter of soil samples was measured by quantifying the weight loss after combustion at 550 °C.

### 2.4. Ecophyisiological, Optical Properties and Color Leaf Measurements

Leaf gas exchange measurements: photosynthesis (A), stomatal conductance (g), intercellular CO_2_ concentration (Ci), transpiration rate (E) and intrinsic water use efficiency calculated as the ratio of photosynthesis rate to transpiration rate (WUE), were measured during a clear sky using a Li-Cor portable photosynthesis system (LiCor 6400, Lincoln, NE, USA) operating at 400 µmol m^−2^ s^−1^ flow rate. Measurements were taken in the morning (10:00 a.m to 12:00 p.m.), according to the protocols of Denaxa et al. [47] and Jifon and Syvertsen [36], on undamaged mature sun leaves located at the central part of the one-year-old shoot of the marked branches, according to Larbi et al. [48]. 

Total directional-hemispherical reflectance of the upper and lower leaf surface was measured with a calibrated spectroradiometer LiCor 1800 (Li-Cor, Nebr, Lincoln, NE, USA) able to scan from 300 to 1100 nm connected to a Li-Cor 1800-12 integrating sphere. To prevent spectral changes due to water losses and metabolic modification, spectral measurements were made immediately after the leaves were picked, according to Baldini et al. [42]. 

Leaves’ colour was measured on the upper surface of one-year leaf using a Konica Minolta CR-400 Chroma Meter (Konica Minolta, Inc., Osaka, Japan) calibrated with a standard white plate at room temperature. The data collected were L* (lightness) and a* (red-green scale) recorded at three random locations on each leaf on twenty leaves collected from the olive trees submitted to different treatment (T, Z and K). 

All leaf f measurements (ecophyisiological, optical properties and color surface) were carried out on 8 July, 24 August and 20 September 2019. 

### 2.5. Olive Analyses and Olive Oils Sensory Evaluation

Considering that the optimal ripening index (RI) for the Correggiolo cultivar is included in the range 2–2.5 of the Jaén index [49], the RI was monitored for each treatment according to the method developed by the Agronomic Station of Jaén defining the RI as a function of fruit colour in both skin and pulp [50]. On the same samples, each olive fruit was examined for the presence of *Bactrocera oleae* infestation, dissecting the fruits to determine the percentage of total infestation (egg, larva or pupa, sting scar, exit holes). Olive water content was gravimetrically determined placing olive samples in oven at 60 °C for 8 days. Olive firmness was determined using a *penetrometer* (PCE-FM 200, PCE Group, Lucca Italy); it was measured at two points on each fruit, and the average readings were reported in g/mm^2^ as exerted pressure.

The total production of the selected trees for each treatment was handpicked; an amount of 50 kg was transformed into oil. Olives were defoliated, washed and milled using a low scale continuous mill (Oliomio^®^; Toscana Enologica Mori, Firenze, Italy) equipped with blade crusher, horizontal malaxator and a two-phase decanter. Olive samples were processed within 24 h of harvest. For each sample the technological settings (temperature (below 27 °C) and the time of malaxation (20 min), the speed of the decanter (4200 rpm) and the flux of water in the separator (0.8 L h^−1^)) were standardized in order to minimize the variability due to the extraction procedures. Oil samples were filtered through cotton filters, poured into dark glass bottles, keeping the headspace to a minimum, and stored in a temperature-controlled cupboard set at 15 ± 1 °C until analysis.

Sensory analyses were carried out by a fully-trained analytical taste panel recognized by the International Olive Oil Council (IOOC) of Madrid and by the Italian Ministry of Agricultural, Food and Forestry Policies. The panel evaluated all oil samples following an incomplete randomized block design. Olive oil samples were placed in blue tasting glasses and the temperature of samples was kept at 15–18 °C. A panel test was established for the present study using a standard profile sheet (IOOC/T20) modified by IBIMET-CNR [51] that allows the obtaining of a more complete description of the organoleptic properties of the oils. The tasters evaluated direct or retronasal aromatic olfactory sensations (olive fruity, green/leaf and secondary positive flavours), gustatory sensations (olive fruity, bitterness and secondary positive flavours) and tactile/kinesthetic sensation (pungency). The tasters had to rate the intensity of the different descriptors on a continuous 0–10 cm scale. Values of the median of sensory data and robust standard deviation were calculated.

### 2.6. Statistical Analysis

The data collected were elaborated using Microsoft^®^ Excel 2007/XLSTAT© (Version 2009.3.02, Addinsoft, Inc., Brooklyn, NY, USA). The significant differences among means at a 5% level were determined by ANOVA followed by a Tukey’s Honestly Significant Difference (HSD) test. Principal component analysis (PCA) has been performed to explore data distribution patterns using physiological data.

## 3. Results and Discussion

### 3.1. ESEM Observations

**Figure 2:**
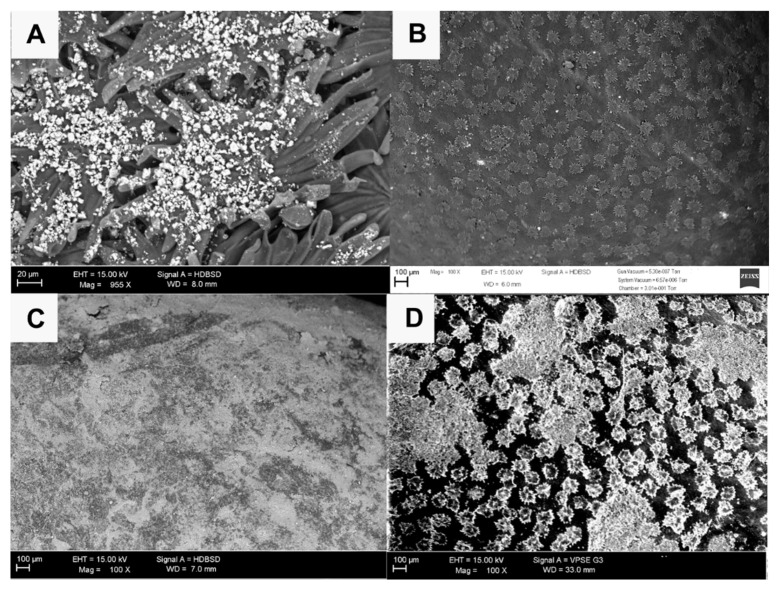
ESEM observations of treated and untreated olive leaves. (**A**) Accumulation of CHA-zeolitite on stellar trichomes that cover the lower surface of the olive leaves; (**B**) Upper surface of test olive leaf; (**C**) Olive leaf treated with kaolin; (**D**) Olive leaf treated with CHA-zeolitite.

Particles of both treatments (K and Z) were more homogenously distributed on the leaves’ surface rather than on the surface of the olive. This higher attachment onto the leaves’ surface is due to their peculiar morphology, characterized by overlapped stellar trichomes, particularly frequent on the lower surface (Figure 2A). 

Since the first foliar application, a good distribution of both K and Z products was observed on the upper surface of the leaves, compared to the test which lacked particles on its surface (Figure 2B). In K treatment, kaolin appeared as a continuous layer and it was not possible to recognize the underlying star hairs (Figure 2C), while in Z treatment, the CHA-zeolitite film was more discontinuous and star hairs were still recognizable (Figure 2D). The same differences were also noted on the lower surface of the leaves. 

This difference in the surface coverage is attributable to both higher amounts of kaolin sprayed at each application compared to the CHA-zeolitite and to the different morphology of kaolin and CHA-zeolitite particles (lamellar vs. pseudo-cubic).

Due to the particular morphology of the olive, characterized by a smooth and curved surface, the adhesion of the particles was less uniform than that observed on the leaves. This difference was observed from the first application and increased in subsequent applications, thanks to the accumulation of the deposited kaolin and CHA-zeolitite particles.

**Figure 3:**
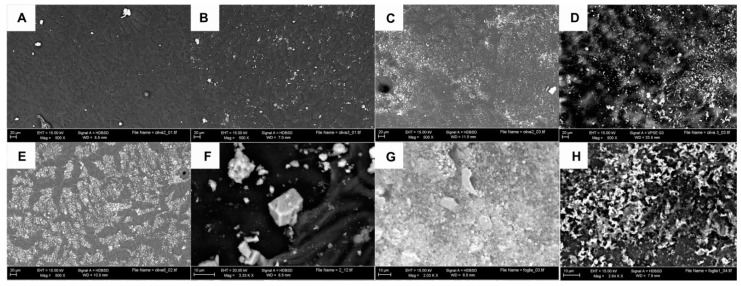
ESEM observations of treated and untreated olives. (**A**) Untreated (T) olive surface; (**B**) Olive surface treated with CHA-zeolitite (Z treatment, first application); (**C**) Olive surface treated with kaolin (K treatment, first application); (**D**) Olive surface treated with CHA-zeolitite (Z treatment, last application); (**E**) Olive surface treated with kaolin (K treatment, last application); (**F**) Morphology of CHA-zeolitite particles; (**G**) Morphology of kaolin particles; (**H**) Olive surface where kaolin appears to be incorporated by waxes.

On the surface of the untreated olives (T), the epicuticular waxes arranged in crystalloid structures (membranous platelets) were well recognizable (Figure 3A), as observed in Carboncella olives by Lanza and Di Serio [43]. Micro-changes of epicuticular waxes, which occur with the progressing of ripening [43], were well visible in the T olives while in K and Z olives these micro-changes were hidden by particle accumulation, especially at the end of the experimentation. 

As reported for the leaves, olives coverage was greater and more homogeneous in K treatment compared to Z treatment (Figure 3B,C); these differences were accentuated due to the accumulation of particles as the experiment progressed (Figure 3D). 

ESEM observations after light rain events highlighted the tendency of K micro-aggregates to disperse and to form a continuous layer (macroscopically visible) on the surface of leaves and olives. With the growth of the olive tissues, the continuous K layer tends to fissure, leaving some areas uncovered (Figure 3E). The different aspect of K and Z films is linked to the specific morphology of the kaolin and CHA-zeolitite particles. CHA-zeolitite particles are mainly pseudo-cubic (Figure 3F) [52] while Kaolin is shaped as sheets/lamellae/irregular flakes (Figure 3G) [53].

Sample observations after heavy rain events showed that both K and Z coatings were well preserved, with the difference that the CHA-zeolitite particles kept their original shape and “anchored” themselves to the waxes, whereas those of kaolin appeared incorporated in waxes (Figure 3H); the same results were observed in apples treated with kaolin [54].

### 3.2. Chemical Analysis on Leaves and Soil Samples

The chemical composition of the soil between the various treatments was very similar in terms of soil organic matter, total N and C, major and trace elements (Supplementary Tables S2 and S3). The study area can be thus considered homogeneous in terms of soil chemistry and N availability to plants. Also, no significant differences were accounted in terms of total C, N and relative isotopic signature of the leaves at the end of the experimentation (Table 1).

### 3.3. Ecophysiological Parameters and Optical Properties

After the first two foliar applications, no significant changes in the photosynthetic rate (A) were observed between the treatments (Table 2). After the 7th application (20th September), a significant decrease of A and stomatal conductance (g) in K plants was observed (Table 2): K plants showed photosynthesis and stomatal conductance values 27% and 55% lower than those of the test plants, respectively. Similar results were found in bean plants by Tworkoski [55], whereas Jifon and Syvertsen [36] observed that the increasing leaf whiteness after kaolin sprays on grapefruit reduced the leaf temperature and increased stomatal conductance and net CO_2_ assimilation rates. At first measurements (8th July), plants belonging to K, Z and T treatments did not show any difference in leaf transpiration (E) but, as the treatments continued (increasing particle accumulation), E decreased significantly in K plants and consequently, the WUE was significantly higher (Table 2). The effect of the kaolin accumulation on physiological parameters is possible to see in the PCA analysis where a clustering of the last date of the kaolin treatment occurs (Supplementary Figure S1). Similar results were observed by Jifon and Syvertsen [36] where WUE in kaolin sprayed leaves of grapefruits was 25% higher than that of control leaves.

No differences were observed in E and WUE between Z and T plants, while g was higher after the last two applications in the Z treatment (22 and 19%, respectively) without, however, influencing the photosynthetic rate (Table 2). Similar results were observed in soybean plants coated with kaolin, where the net radiation was reduced by 8% and short-wave irradiation was reduced by 20%, suggesting a potential reduction in transpiration and water use [34]. Also, Le Grange [56] reported a reduction in photosynthetic rates in kaolin sprayed leaves attributable to increased reflection and absorption of light reduced by 20–40%. Some authors [57,58] reported that kaolin treatment did not reduce the photosynthesis of single leaves but increased the photosynthesis of the whole canopy and therefore the productivity. In rainfed olive trees, Brito et al. [59] demonstrated that kaolin treatment counteracted the effect of water shortage and high light intensity on leaf sclerophyll and on stomatal density. Still in rainfed olive orchards, kaolin application contributed to keep a better water status by creating a specific microclimate around the leaves; moreover, it alleviated the adverse effect of summer stress through distinct physiological and biochemical responses [59].

In our study, the positive effect of kaolin was not observed because the olive trees are grown in environmental conditions (high rainfall and low temperatures) that do not lead to stress conditions; on the contrary, the abundant covering of the kaolin film had a negative effect on photosynthesis, that decreased during the delicate ripening phase of the olive fruits. Stomatal conductance and transpiration were also significantly reduced in K trees at the end of the experiment. This was probably the result of the abundant accumulation of kaolin on the leaf surfaces, leading to obstruction of stomata, with an alteration of leaf gas exchanges.

The authors are aware of the fact that, in these environmental conditions, a lower amount of kaolin or less frequent applications would have been sufficient (the concentration of kaolin was five-fold higher than that of CHA-zeolitite), but we aimed at reproducing the operative protocols commonly adopted for olive fruit fly defense. In the several Italian regions where olive cultivation is practiced, indeed, standardized protocols for protection from the olive fruit fly are used, regardless of the different climate conditions. A differentiation for kaolin-based treatments would be necessary and specific protocols should be developed for each different cultivation environment. These protocols must guarantee an adequate level of defense against fly attacks without significantly altering the physiological parameters of the plant.

Since the 4th application, a significant increase in Ci (CO_2_ inside the lamina) was observed in Z plants compared to the other treatments. It has been reported that zeolites can adsorb carbon dioxide molecules and release them slowly into the environment; also, it has been suggested that when zeolites are spread on plant leaves, they may increase the amounts of CO_2_ near the stomata, concomitantly increasing the photosynthesis rate [60]. In our experiment, however, we have observed no significant effect on the photosynthesis rate in Z plants. On the contrary, K leaves showed lower Ci that is in agreement with the observed decrease in A. Farquhar and Sharkey [61] indeed asserted that where CO_2_ diffusion limits A, a decrease in Ci would also occur. 

**Figure 4:**
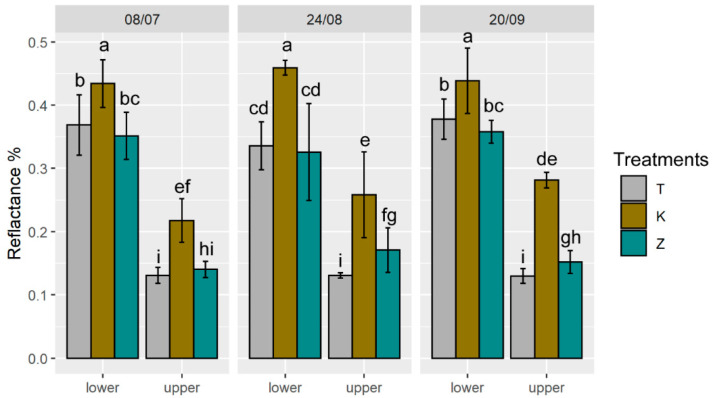
Mean reflectance between 400 and 700 nm measured on the lower and upper part of the leaves of T (control), K (kaolin) and Z (CHA-zeolitite) treatments. Error bars represent standard deviation. Different letters above the bars for each group of histograms indicate significant differences according to ANOVA and Tukey’s HSD test (*p* < 0.05).

In our study, the upper and lower sides of K leaves showed a significant increase in reflectivity compared to the other treatments at all dates (Figure 4). The reflectance is the ability to reflect part of the incident light on a given surface and its effectiveness in reflecting radiant energy. Similar results were observed in grapefruit leaves coated with kaolin, which showed a higher reflectance compared to control leaves [36]. 

In Z leaves, the reflectance was similar to the T during the summer; only after the last application (29th September) did it increase (Figure 4). The different reflective capacity of the two films is attributable both to the different colour of the powders, white for kaolin (higher light reflectance) and light brown for CHA-zeolitite (lower light reflectance) and to the morphology of the particles, lamellar for kaolin (higher reflectance) vs. pseudo-cubic for CHA-zeolitite (lower reflectance). Furthermore, leaf reflectance data (Figure 4) showed that there is no difference when the number of treatments increases.

**Figure 5:**
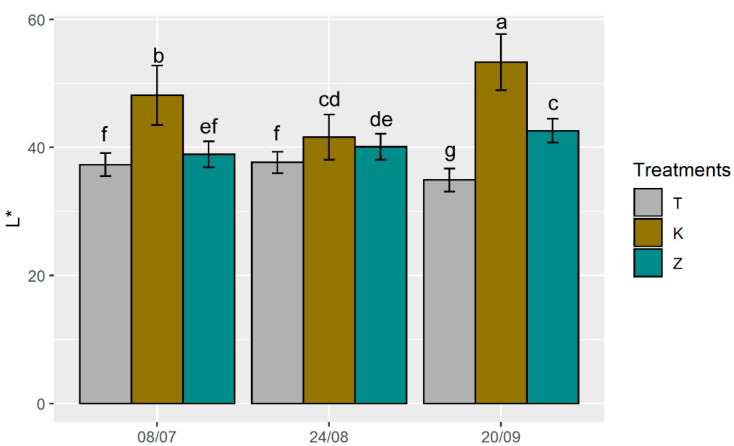
Changes in L*color values measured on the upper part of the leaves of T (control), K (kaolin) and Z (CHA-zeolitite) treatments. Error bars represent standard deviation. Different letters above the bars for each group of histograms indicate significant differences according to ANOVA and Tukey’s HSD test (*p* < 0.05).

**Figure 6:**
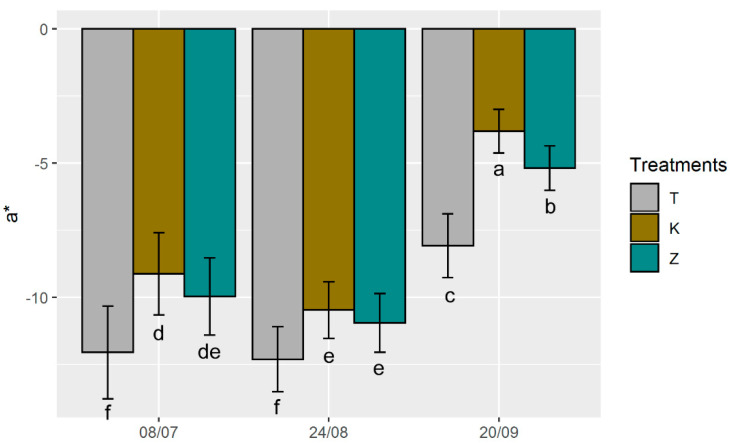
Changes in a*colour values measured on the upper part of the leaves of T (control), K (kaolin) and Z (CHA-zeolitite) treatments. Error bars represent standard deviation. Different letters above the bars for each group of histograms indicate significant differences according to ANOVA and Tukey’s HSD test (*p* < 0.05).

Colour measurement carried out on leaves treated with Kaolin showed a greater lightness (L*) compared to the test and CHA-zeolitite leaves at all dates (Figure 5). This difference in L* value between kaolin and CHA-zeolitite is due to the different conformation and colour of the kaolin (phyllosilicate) and natural zeolite (tectosilicate). After the 4th foliar application (24th August), the L* value of K leaves was greater by 22% and 19% with respect to those of T and Z leaves, respectively. Colour measurements performed on K leaves after the 3rd application showed lower L* values than the previous measurements, while no differences were accounted in T and Z treatments. The lower L* values observed in K treatment were probably due to kaolin leaching due to rainfall occurring during the previous days (Supplementary Figure S1). At the last measurement (performed on the 20th September), L* values were higher in both K and Z treatments compared to the T, suggesting that an accumulation of both kaolin and CHA-zeolitite on leaves occurred. Our data agrees with Jifon and Syvertsen’s [36] measurements on grapefruit leaves treated with kaolin. Regarding the a* value, in the first two measurements T leaves showed higher values than those recorded in K and Z treatments, that were similar (Figure 6). It is interesting to note that contrary to L*, the a* value was not affected by the rainfall. After the 3rd measurement, a* decreased in all the treatments; this was probably caused by the leaf seasonality, since a reduction in the chlorophyll content in the leaf is expected at the end of the hot season [62].

### 3.4. Olive Analyses and Olive Oil Sensory Evaluation

In a year characterized by lower temperature (2019) (Supplementary Figure S2) with a high risk posed by the olive fruit fly, zeolite and kaolin sprays have significantly reduced the incidence of *Bactrocera oleae*; in fact, olives produced by Z and K olive trees present a decrease (over 40%) of infestation compared to control (Table 3). Water content of olive treated with kaolin was higher than the water content of olive treated with zeolitite while the olive from untreated trees showed similar value to both treatments (Table 3). The olive firmness did not statistically differ within the treatments, and the same results were observed in pear fruits treated with kaolin [63].

**Figure 7:**
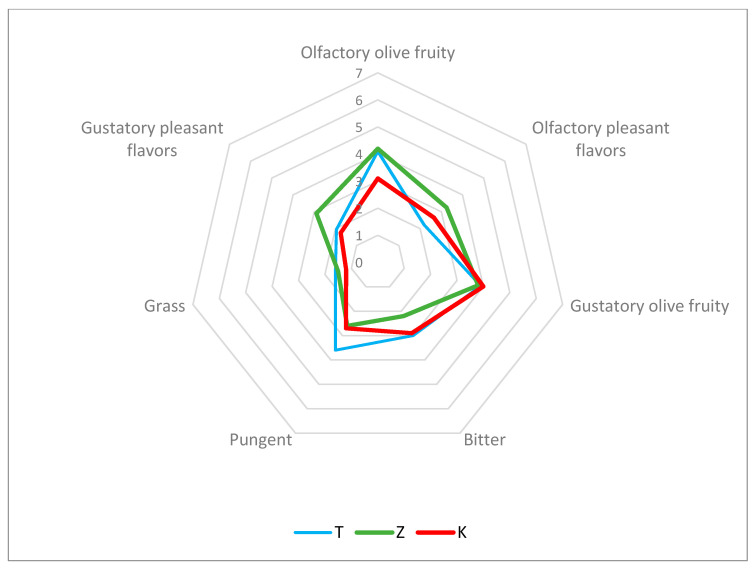
Sensory profiles of olive oils produced by plants treated with CHA-zeolitite (Z), Kaolin (K) and untreated plants (T).

**Figure 8:**
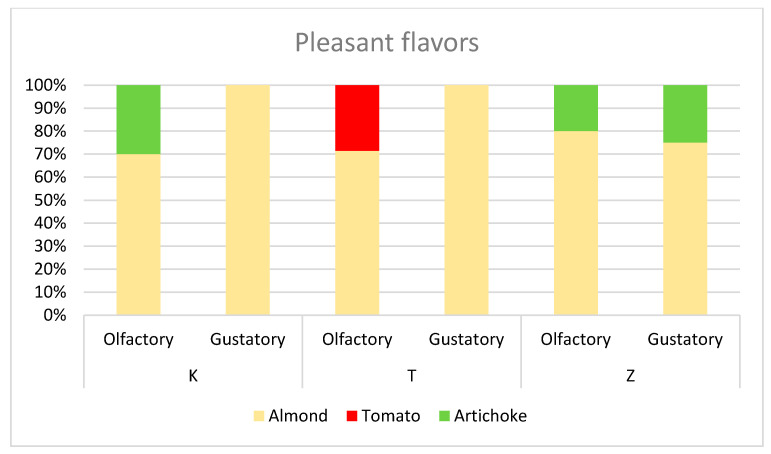
Pleasant flavors of olive oils produced by plants treated with CHA-zeolitite (Z), Kaolin (K) and untreated plants (T).

The sensory profiles of olive oils extracted from plants treated with Kaolin (K), CHA-zeolitite (Z) and control (T) are shown in Figure 7. On a sensory level, the differences found in the oils were slight: Z and T olive oils showed a higher intensity of olfactory olive fruity than K olive oil. For the hint of bitterness K and T olive oil showed higher intensity than Z while for the hint of pungency test olive oil had higher intensity compared to K and Z olive oil. Test oil showed a lower intensity in olfactory secondary flavours while Z oil had a higher intensity in both olfactory and gustatory secondary flavours. Detailed examination of the pleasant flavours (Figure 8) revealed that, at the olfactory level, oils produced from both treatments had an artichoke scent and were perceived as fresher with respect to the oils produced by the test, that smelled of ripe tomato.

At the gustatory level, no differences were observed between the T and K oils, for which the tasters only perceived the hint of green almond. On the other hand, it is interesting to underline that the oil produced from olive trees treated with zeolite presented, in addition to the prevailing hint of almond, a note of artichoke which gave the oil a note of freshness compared to others.

De la Roca [30] found that kaolin application against the olive fruit fly significantly reduced the percentage of infested olives. Saour and Makee [31] showed that a kaolin-based particle film formulation significantly reduced fruit infestation levels; the authors hypothesized that adult flies may fail to recognize kaolin sprayed olive trees, and the gravid females are repelled due to the tactile unsuitable surface texture of particle film-treated olives.

## 4. Conclusions

In the scenario of sustainable and environmentally friendly olive oil production, both treatments represent valid alternatives to chemical insecticide. From an economic point of view, CHA-zeolitite represents an advantage because the recommended application rate is five times lower than that commonly used for kaolin. Moreover, CHA-zeolitite volcanic rocks abound in Central Italy and are already exploited for several purposes, including the production of micronized powder from the granular material resulting from building block cutting in quarries. CHA-zeolite supplying is thus relatively less impactful from an environmental point of view, with respect to other types of powders that are quarried and manufactured in foreign countries.

ESEM observation performed on leaf and olive surfaces highlighted microstructural differences between the two tested particle films which influenced some ecophysiological parameters. The intercellular CO_2_ concentration was positively influenced by CHA-zeolitite application while kaolin application decreased photosynthesis, stomatal conductance and transpiration rates compared to the other foliar treatments. Therefore, in hot environments, the use of kaolin has the dual function of protecting the olive tree both from high temperatures and from the olive fly but the resulting impactful coating caused a reduction of photosynthesis that can, however, be compensated by an increase in WUE due to the reduced transpiration. The continuous layer of kaolin on leaf surface has also significantly influenced the leaf reflectance thanks to its crystal morphology, colour and application rate. 

In a cold and humid environment (such as our experimental conditions), CHA-zeolitite was found to be the ideal compound because it exerted a protective effect against olive fruit fly attack, similar to kaolin, but left the leaf gas exchanges unaltered. Moreover, oils obtained from CHA-zeolitites showed higher intensities of gustatory and olfactory pleasant flavours than olive oils produced from kaolin and untreated trees, thus enhancing the quality and sustainability characteristics of this product.

## Figures and Tables

**Table 1:** Results of leaves analysis (EA-IRMS) from each experimental plant treated with kaolin (K), CHA-zeolitite (Z) and the control (T). TN and TC are the total nitrogen and carbon content measured by EA analysis, δ15N and δ13C are the isotopic signatures expressed as delta notation by IRMS. Values are expressed as the mean of three replicates ± standard deviation. The same letters in the same column express no significant differences (*p* > 0.05) as results of ANOVA and Tukey’s (HSD) tests.

Treatment	TN (%)	TC (%)	*δ^15^N* (‰)	*δ^13^C* (‰)
T	1.47 ± 0.02 a	45.51 ± 1.12 a	−3.71 ± 2.06 a	−27.76 ± 0.28 a
K	1.69 ± 0.17 a	47.71 ± 2.03 a	−0.53 ± 1.49 a	−28.59 ± 0.42 a
Z	1.44 ± 0.36 a	47.05 ± 3.39 a	−2.06 ± 1.71 a	−28.43 ± 0.37 a

**Table 2:** Ecophysiological parameters measured after each foliar application of K (kaolin), Z (CHA-zeolitite), and T (control). Data are presented as mean ± standard deviation. Different letters (a,b,c) indicate significant differences according to ANOVA and Tukey’s HSD test (*p* < 0.05) at each application date.

Application Date	Treatment	A ^1^ μmol CO_2_ m^−2^ s^−1^	G ^2^ mmol m⁻² s⁻¹	Ci ^3^ μmol CO_2_ mol air	E ^4^ mol H_2_O m^−2^ s^−1^	WUE ^5^
8 July	K	13.03 ± 0.75 a	0.39 ± 0.02 a	321.98 ± 4.05 a	9.68 ± 0.46 a	1.38 ± 0.09 a
	T	13.14 ± 1.05 a	0.32 ± 0.02 a	309.49 ± 4.89 a	9.82 ± 0.48 a	1.34 ± 0.09 a
	Z	12.14 ± 0.85 a	0.33 ± 0.04 a	309.36 ± 6.20 a	9.06 ± 0.71 a	1.42 ± 0.11 a
24 August	K	9.98 ± 0.64 a	0.20 ± 0.02 b	287.65 ± 5.02 b	8.01 ± 0.61 b	1.28 ± 0.07 a
	T	11.93 ± 0.78 a	0.28 ± 0.03 a,b	295.35 ± 4.57 b	10.28 ± 0.67 a	1.18 ± 0.06 a
	Z	12.35 ± 0.59 a	0.34 ± 0.02 a	312.77 ± 3.51 a	9.46 ± 0.58 a,b	1.37 ± 0.1 a
20 September	K	9.5 ± 0.55 b	0.12 ± 0.01 c	248.86 ± 6.94 c	2.91 ± 0.23 b	3.36 ± 0.15 a
	T	13.03 ± 0.49 a	0.28 ± 0.01 b	299.98 ± 2.06 b	5.55 ± 0.21 a	2.36 ± 0.06 b
	Z	12.19 ± 0.76 a	0.33 ± 0.01 a	317.69 ± 2.59 a	5.99 ± 0.20 a	2.02 ± 0.08 b

^1^ A is the net photosynthetic rate; ^2^ g is stomatal conductance; ^3^ Ci is the intercellular CO_2_ concentration; ^4^ E is the transpiration; ^5^ WUE is the water use efficiency calculated as the ratio of photosynthesis rate to transpiration rate.

**Table 3:** Ripening index (RI), percentage of olive fruit fly infestation, water content and fruit firmness in olive from trees treated withK (kaolin), Z (CHA-zeolitite), and T (control). Data are presented as mean ± standard deviation. Different letters in the same column (a,b,c) indicate significant differences according to ANOVA and Tukey’s HSD test (*p* < 0.05).

Treatment	RI	% Infestation	H_2_O (%)	Firmness ^1^
K	2.6	26	46.5 ± 1.4 a	55.0 ± 28.9
T	2.58	70	43.2 ± 0.6 b	52.7 ± 29.9
Z	2.48	34	44.7 ± 0.4 a,b	48.7 ± 28.8
*p*-value	/	/	0.038	ns

^1^ express as g/mm^2^.

## Supplementary Materials

The following are available online at https://www.mdpi.com/article/10.3390/foods10061291/s1, Supplementary Table S1: Mineralogical composition (XRPD Rietveld-RIR method) of the kaolin and CHA-zeolitite supplied by Balco s.p.a and used in the experimentation. Data from the product’s technical sheet supplied by the company; Supplementary Table S2: Results of soil analysis (oven-combustion, EA-IRMS) from each experimental plant treated with kaolin (K), CHA-zeolitite (Z) and the control (T); Supplementary Table S3: Results of soil analysis (2 replicates) by X-Ray Fluorescence (XRF) from each experimental plant treated with kaolin (K 1 and 2), CHA-zeolitite (Z 1 and 2) and the control (T 1 and 2); Supplementary Figure S1: PCA of the ecophysiological parameters measured after the foliar applications of K (kaolin), Z (CHA-zeolitite), and T (control); Supplementary Figure S2: Minimum, mean and maximum temperature (°C) and rainfall (mm) recorded in the period 1st Julay-31st October.
