# Supplementary material for: Distinct Particle Films Impacts on Olive Leaf Optical Properties and Plant Physiology
Source: Foods. 2021 Jun 4;10(6):1291. doi: 10.3390/foods10061291 (PMC8228084; doi:10.3390/foods10061291)
Supplement: Supplementary file 1 [file foods-10-01291-s001.zip › supplementary material.pdf]

Supplementary table S1.

Supplementary table 1. Mineralogical composition (XRPD Rietveld-RIR method) of the kaolin and CHA-zeolitite supplied by Balco s.p.a and used in the experimentation. Data from the product's technical sheet supplied by the company

**Mineralogical composition (%)**	**Kaolin**	**CHA-zeolitite**
Kaolinite	87	0
Quartz	3	0
Muscovite	4	0
Chabazite	0	65
Phillipsite	0	3
K-feldspar	0	5
Biotite	0	2
Pyroxene	0	3
Volcanic glass	0	22
Others	6	0
Total Zeolitic content	0	68

Supplementary table 2: Results of soil analysis (oven-combustion, EA-IRMS) from each experimental plant treated with kaolin (K), CHA-zeolitite (Z) and the control (T). SOM is the soil organic matter measured by combustion at 550 °C (mean of 2 replicates), TN and TC are the total nitrogen and carbon content measured by EA analysis, δ15N and δ13C are the isotopic signature expressed as delta notation by IRMS (values are expressed as mean of 3 replicates). Standard deviation within brackets. Same letters express no significant differences (p>0.05) as results of ANOVA and TUKEY (HSD) tests.

	T	K	Z
%			
SOM	7.90	8.45	7.95
TN	0.29 (0.02)[a]	0.31 (0.04)[a]	0.27 (0.05)[a]
TC	5.11 (0.45)[a]	5.06 (0.14)[a]	4.93 (0.18)[a]
‰			
$\delta^{15}N$	+2.17 (0.77)[a]	+2.79 (0.62)[a]	+1.29 (1.28)[a]
$\delta^{13}C$	-17.57(1.08)[a]	-18.12 (0.37)[a]	-17.31 (1.05)[a]

Supplementary table 3: Results of soil analysis (2 replicates) by X-Ray Fluorescence (XRF) from each experimental plant treated with kaolin (K 1 and 2), CHA-zeolitite (Z 1 and 2) and the control (T 1 and 2). LOI represent the loss on ignition (volatile losses at 1000°C).

	T 1	T 2	K 1	K 2	Z1	Z 2
%						
**SiO_2**	50.87	53.76	52.62	52.56	50.72	51.10

**TiO_2**	0.54	0.50	0.58	0.54	0.52	0.55
**AL_2O_3**	12.59	12.15	13.29	12.61	12.65	12.64
**Fe_2O_3**	4.25	4.00	4.45	4.10	4.20	4.31
**MnO**	0.11	0.11	0.11	0.11	0.12	0.11
**MgO**	2.51	2.34	2.55	2.48	2.50	2.47
**CaO**	9.77	9.12	8.38	8.63	9.78	9.32
**Na_2O**	0.85	0.94	0.85	0.85	0.87	0.86
**K_2O**	2.27	2.33	2.43	2.32	2.28	2.27
**P_2O_5**	0.18	0.18	0.20	0.21	0.16	0.19
**LOI**	16.06	14.57	14.53	15.59	16.19	16.19
**Total**	100.0	100.0	100.0	100.0	100.0	100.0
ppm						
**Ba**	393	385	410	402	399	398
**Ce**	39	36	40	40	40	42
**Co**	13	13	14	12	12	13
**Cr**	90	89	97	90	87	90
**Cu**	73	57	57	73	59	67
**Ga**	15	14	16	15	15	17
**Hf**	3	3	3	3	5	4
**La**	12	9	15	10	13	15
**Nb**	12	11	12	12	11	12
**Nd**	26	18	23	23	18	23
**Ni**	66	61	65	62	61	62
**Pb**	32	33	34	37	27	32
**Rb**	95	100	106	100	93	98
**Sc**	9	8	9	8	10	10
**Sr**	295	279	273	269	294	289
**Th**	6	5	6	5	5	6
**V**	81	72	83	78	78	84
**Y**	19	19	20	18	19	19
**Zn**	75	76	86	81	67	74
**Zr**	185	160	167	156	195	179

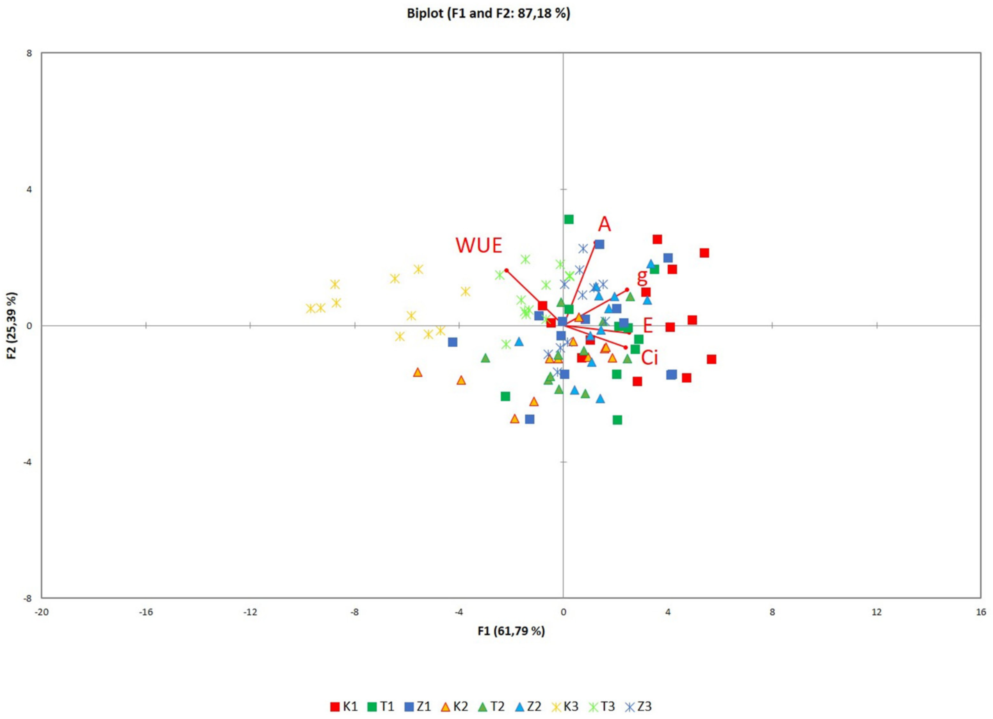

Supplementary figure 1. PCA of the ecophysiological parameters measured after the foliar applications of K (kaolin), Z (CHA-zeolitite), and T (control).

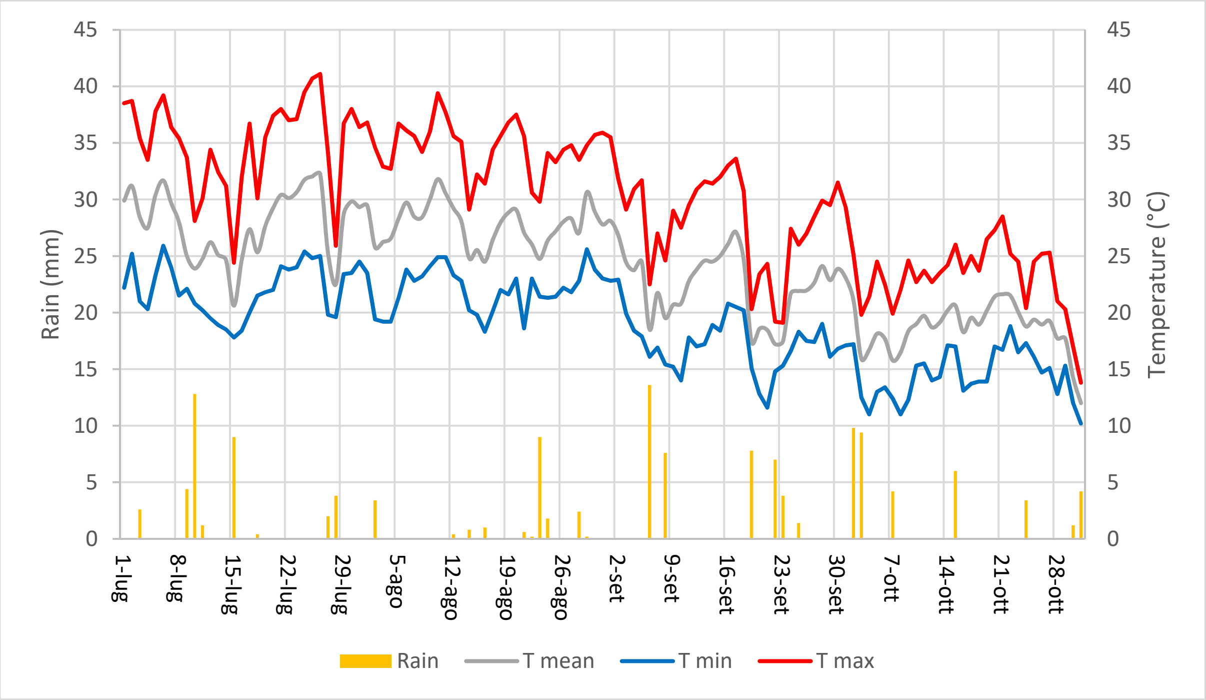

Supplementary figure 2. Minimum, mean and maximum temperature (°C) and rainfall (mm) recorded in the period 1st Julay-31st October.
