# Supplementary figures and images for: Distinct Particle Films Impacts on Olive Leaf Optical Properties and Plant Physiology
Source: Foods. 2021 Jun 4;10(6):1291. doi: 10.3390/foods10061291 (PMC8228084; doi:10.3390/foods10061291)

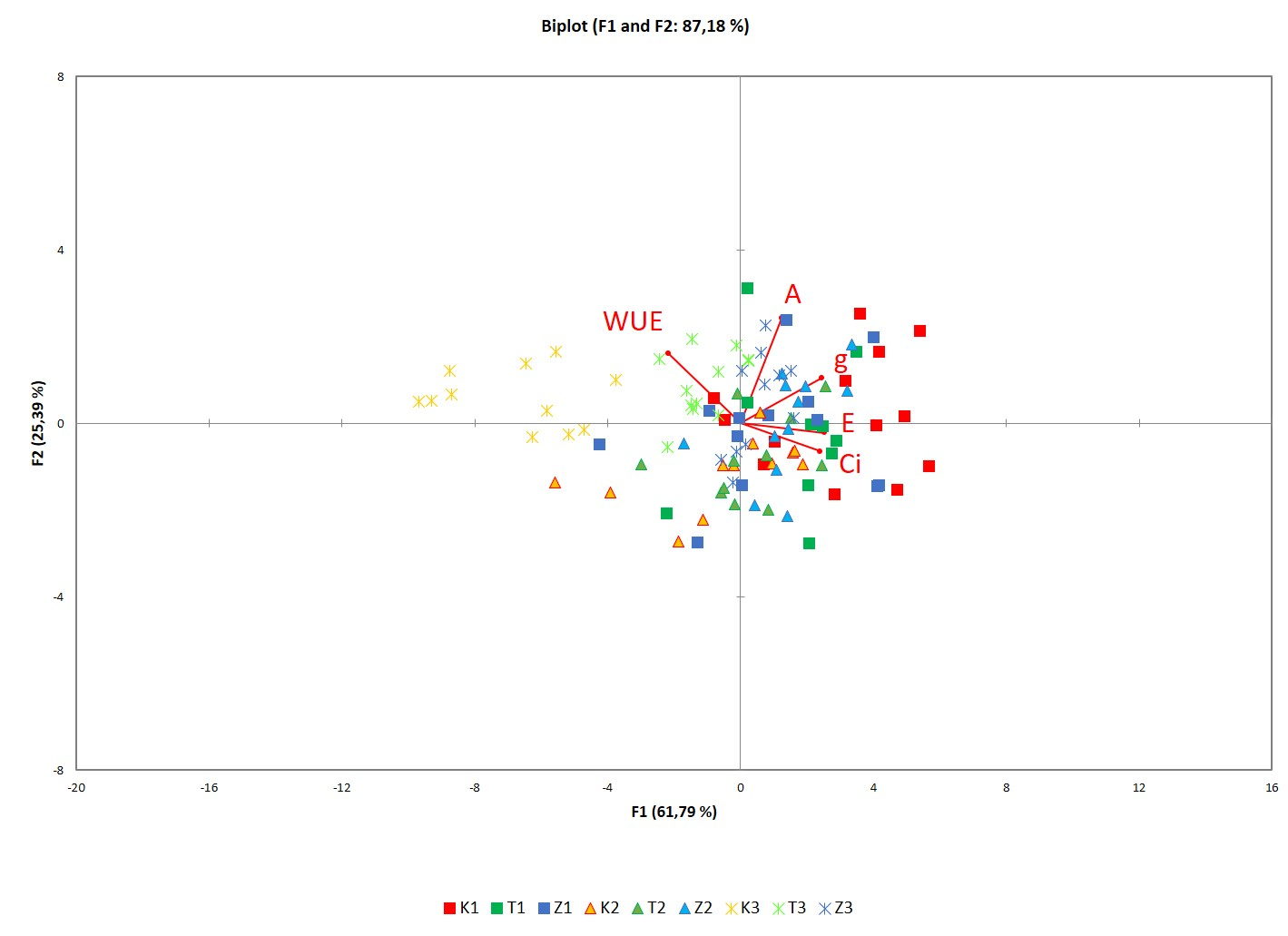

Supplement: Supplementary file 1 [file foods-10-01291-s001.zip › supplementary1.tif]

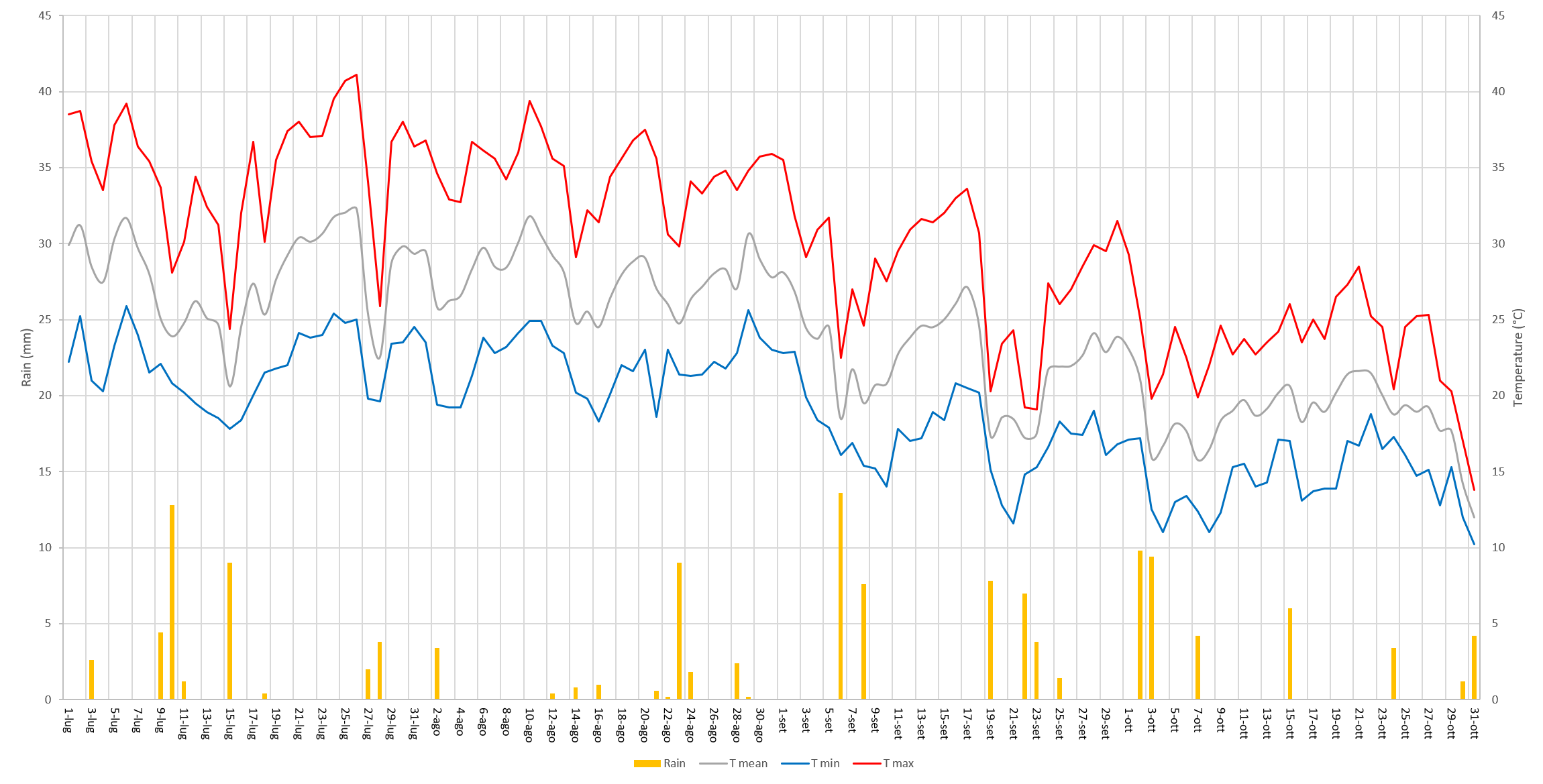

Supplement: Supplementary file 1 [file foods-10-01291-s001.zip › supplementary2.tif]
